# Beyond diagnostic categories: sensory processing as a transdiagnostic mechanism in autism, ADHD, and PTSD – a neuropsychoanalytic conceptual analysis

**DOI:** 10.3389/fpsyg.2026.1871344

**Published:** 2026-07-17

**Authors:** Betul Sağlam

**Affiliations:** Department of Psychology, Istanbul Commerce University, Istanbul, Türkiye

**Keywords:** ADHD, alpha function, autism, neuropsychoanalysis, prenatal stress, procédés auto-calmants, psychic transparency, PTSD

## Abstract

The post-pandemic surge in adult ADHD presentations has intensified a question that categorical diagnosis tends to foreclose: What do autism, ADHD, and PTSD share at the level of developmental organization? This paper offers a conceptual analysis of sensory processing differences as a transdiagnostic mechanism linking these three conditions, examined through a neuropsychoanalytic lens. Drawing on current empirical literature and the French psychosomatic tradition, we explore how the sensory modalities most consistently implicated across all three (auditory, tactile, and vestibular) are precisely those that achieve functional maturity *in utero*, rendering their organization vulnerable to the quality of the prenatal sensory environment. Maternal prenatal stress, mediated through HPA axis activation and epigenetic programming of the NR3C1 glucocorticoid receptor gene, constitutes one documented pathway through which this vulnerability may be established. We further examine how Bydlowski’s concept of psychic transparency during pregnancy, in which the usual defenses against unconscious material are lowered and archaic, unprocessed material resurfaces, may provide a psychoanalytic account of intergenerational beta element transmission, whose somatic correlate is the molecular inscription documented by prenatal stress research. The self-soothing and self-regulatory behaviors observed across these three populations are reconsidered not as symptoms to be eliminated but as proto-alpha mechanisms: bodily attempts at psychic organization where adequate containment has been unavailable. Clinical and research implications of this conceptual framework are discussed.

## Introduction

The years following the COVID-19 pandemic have witnessed a striking increase in clinical referrals centered on a single question: Could I have ADHD? Population-based data confirm this is not merely anecdotal. In British Columbia, the monthly incidence of newly diagnosed adult ADHD rose from 8.8 per 100,000 pre-pandemic to 34.8 post-pandemic (a nearly fourfold increase), with a disproportionate rise among young adult females and, notably, among individuals with prior histories of stress-related disorders (Hu et al., 2025). Presenting complaints are remarkably consistent: difficulty sustaining attention, forgetfulness, and an inability to follow through on tasks; yet the same individuals report hours of absorbed, effortless engagement with activities that capture their interest. This clinical picture raises two questions that are rarely asked together. The first is descriptive: Do these individuals meet DSM-5 criteria for ADHD? The second, and more pressing, is etiological: Why now, and why with such uniformity of presentation?

According to DSM-5, an ADHD diagnosis in adults requires at least five symptoms of inattention and/or hyperactivity-impulsivity, onset before age 12, cross-situational impairment, and the exclusion of alternative explanations (American Psychiatric Association [APA], 2013). Global meta-analytic estimates place the prevalence of persistent adult ADHD between 2.58% and 4.61%, with symptomatic presentations reaching 6.76% when broader criteria are applied (Song et al., 2021). These figures likely underestimate true prevalence, particularly in females. Women with ADHD more often present with internalizing symptoms, develop compensatory strategies, and receive prior diagnoses of anxiety or depression before ADHD is identified (Morgan, 2024). This pattern of camouflaging (the conscious and unconscious masking of neurodivergent traits) carries a cumulative psychosocial cost; diagnosis in adulthood is frequently accompanied by grief over years lived without adequate support (van der Putten et al., 2024; Morgan, 2024). Increased awareness and reduced stigma may therefore partly explain the post-pandemic surge in help-seeking, particularly among adult women.

The same pandemic that drove this wave of ADHD referrals also produced elevated rates of PTSD in the general population. A meta-analysis of 76 studies encompassing 106,713 individuals found a pooled prevalence of post-traumatic stress symptoms of 28.34% among those exposed to pandemic-related trauma (Qiu et al., 2021), with estimates varying substantially across countries and measurement instruments depending on sampling method and diagnostic criteria applied (Catapano et al., 2025). This co-occurrence raises a specific clinical problem: PTSD and ADHD share a remarkably similar surface presentation. Both are characterized by attentional difficulties, emotional dysregulation, hyperarousal, and heightened sensitivity to sensory stimulation; the two conditions are frequently mistaken for one another in clinical settings, and each may mask or exacerbate the other when they co-occur (Wendt et al., 2023). The post-pandemic surge in adult presentations seeking help for attentional difficulties may therefore reflect not a single diagnostic entity but a convergence of distinct conditions whose surface phenomenology overlaps. This clinical ambiguity prompted the question at the heart of this paper: If conditions as etiologically different as ADHD and PTSD can produce such similar presentations, what common mechanism might account for this convergence?

These observations leave a more fundamental question unanswered. The clinical picture of attentional dysregulation, emotional lability, and sensory overreactivity is not unique to ADHD. The same features appear, with striking regularity, across autism spectrum conditions and PTSD, three categories that DSM-5 treats as distinct but that share a transdiagnostic dimension that has received insufficient theoretical attention: sensory processing differences (Scheerer et al., 2024; Jurek et al., 2025; Fleming et al., 2024). This paper offers a conceptual analysis of that shared dimension. Drawing on current empirical literature and on psychoanalytic theory, particularly the French psychosomatic tradition, we examine how sensory processing differences across these three diagnostic categories may be understood as expressions of a common developmental organization, rooted in prenatal sensory experience and shaped by early relational conditions. We further explore what this reading offers to an understanding of the self-soothing and self-regulatory behaviors observed in these populations, and what clinical implications follow from reconsidering them as proto-alpha mechanisms rather than pathological symptoms.

## Sensory processing: definition, neural substrates, and clinical manifestations

Sensory processing refers to the neurological capacity to receive, regulate, and integrate information from both the external environment and the internal body, generating adaptive behavioral, emotional, and motor responses (Ayres, 1972; Miller et al., 2007). Beyond the classical five senses, this encompasses the vestibular system, proprioception, and interoception (the perception of internal physiological signals including heartbeat, hunger, and bodily tension) (Tsakiris et al., 2011; Harricharan et al., 2021). Sensory processing is not localized to a single brain region; it depends on the coordinated activity of subcortical and cortical structures, including the thalamus, basal ganglia, superior temporal sulcus, parietal areas, and prefrontal cortex, whose integrative work enables the organism to filter, prioritize, and assign meaning to incoming sensory data (Roley et al., 2007).

When this integrative function is disrupted, sensory processing differences emerge, characterized by atypical responses to stimulation: hypersensitivity, hyposensitivity, sensory seeking, or low registration (a reduced responsiveness to sensory input in which higher levels of stimulation are required before the nervous system registers and responds; Jurek et al., 2025). These patterns are not mutually exclusive and frequently co-occur across different modalities in the same individual. Their consequences extend well beyond the sensory domain; sensory processing differences are consistently associated with emotion dysregulation, attentional difficulties, and social withdrawal across neurodevelopmental populations (Brandes-Aitken et al., 2024; Chen et al., 2024).

In autism spectrum conditions, sensory differences are now formally recognized within DSM-5 as a core diagnostic feature, incorporated in 2013 under the domain of restricted and repetitive behaviors (American Psychiatric Association [APA], 2013). Up to 90% of autistic individuals experience clinically significant sensory processing differences, with auditory and tactile modalities most consistently documented (Schaaf and Puts, 2023). This distribution carries developmental significance, as we explore below: both systems achieve functional maturity *in utero*, well before the visual system’s predominantly postnatal organization. At the neurobiological level, autistic sensory profiles are associated with atypical maturation of auditory cortex response latencies, disrupted thalamocortical connectivity, and white matter microstructural differences in posterior sensory regions, changes observable in high-risk infants before behavioral symptoms appear (Nagode et al., 2017).

In ADHD, sensory processing differences have historically been treated as secondary to attentional dysregulation rather than as a distinct clinical dimension. Recent meta-analytic evidence challenges this. Across 30 studies encompassing 5,374 participants, individuals with ADHD showed significantly elevated atypical sensory processing across all four primary domains (sensitivity, avoidance, seeking, and low registration) compared to controls, with moderate to large effect sizes across auditory, visual, tactile, and oral modalities (Jurek et al., 2025). Low registration severity increased with age, suggesting that sensory processing differences in adult ADHD are not residual childhood features but a persisting, potentially amplifying dimension of the condition. A parallel investigation found that across sensory subtypes in both autism and ADHD, whether over-responsivity, under-responsivity, or sensory seeking, emotion dysregulation scores were elevated, pointing to a shared link between sensory and affective dysregulation that operates independently of diagnostic category (Brandes-Aitken et al., 2024).

In PTSD, the relevance of sensory processing has gained empirical traction, though it remains undertheorized relative to the disorder’s canonical fear-circuit model. It should be noted explicitly that PTSD is not a neurodevelopmental condition in the conventional sense; its onset follows a discrete traumatic event rather than reflecting early organizational differences. We include it here precisely because of that contrast: that a developmentally acquired condition produces sensory dysregulation phenotypically indistinguishable from that of neurodevelopmental origin raises a fundamental question about the substrate on which trauma acts. Fleming et al. (2024) summarize converging evidence that PTSD is associated with structural and functional variability in primary sensory cortices, and that this variability correlates with symptom severity independently of threat-processing circuits. Of particular theoretical importance is the susceptibility hypothesis these authors advance: pre-existing alterations in sensory cortex organization may render certain individuals more vulnerable to developing PTSD following trauma exposure, rather than the alterations being solely its consequence. Harricharan et al. (2021) further demonstrate that PTSD disrupts the integration of both exteroceptive and interoceptive signals, with sustained hyperactivation of the innate alarm system producing a state in which body and environment are processed through a persistent filter of threat. In the dissociative subtype, this pattern inverts into sensory deafferentation (a cortical suppression of somatic input mediated by thalamocortical gating mechanisms and prefrontal inhibitory activity, producing subjective disconnection from bodily experience; Harricharan et al., 2021) whose developmental implications are taken up in the sections that follow.

The shared phenomenology across these three clinical pictures (compromised sensory filtering, downstream consequences for emotional regulation, and persistent self-regulatory difficulties) has been confirmed transdiagnostically: sensory phenotypes cluster similarly across autism and ADHD regardless of diagnostic label, and sensory processing differences predict behavioral outcomes over and above categorical membership (Scheerer et al., 2024). What this literature has not addressed is the developmental question at the center of this analysis: How might such shared regulatory patterns arise, and what does a psychoanalytic reading of their origins contribute to clinical understanding?

## Before the self: prenatal sensory organization and the origins of regulatory capacity

Underlying the transdiagnostic convergence described above is a question that clinical and neuroscientific accounts rarely pose: Where does sensory processing begin? The implicit assumption in most frameworks is that sensory organization is a postnatal phenomenon, shaped by experience following birth. The developmental record suggests otherwise.

Sensory systems do not become functional simultaneously. The tactile system is the first, with responses to cutaneous stimulation observable as early as the seventh to eighth gestational week (Fagard et al., 2018). The vestibular system begins structural differentiation by the sixth week of gestation and achieves functional maturity by the end of the second trimester, continuously stimulated by maternal movement throughout intrauterine life (Roley et al., 2007). The auditory system becomes responsive to external sound between the twenty-fifth and twenty-eighth gestational weeks; the fetus demonstrates preferential responses to the maternal voice, a recognition that persists into the neonatal period, indicating that early auditory learning occurs before birth (Fagard et al., 2018). The visual system, by contrast, receives minimal meaningful stimulation *in utero* and matures predominantly postnatally. The sensory modalities most consistently implicated in autism and ADHD, namely auditory and tactile, are precisely those that organize earliest, within the prenatal environment. Their atypicality may reflect not only postnatal experience but the quality of the intrauterine sensory matrix in which they first became functional.

This observation is supported by the literature on maternal prenatal stress. Lautarescu et al. (2020) summarize two decades of evidence that maternal psychological stress during pregnancy is causally associated, across large population cohorts controlling for postnatal confounders, with increased risk of emotional, behavioral, and cognitive problems in offspring, including ADHD and anxiety. The primary mediator is the hypothalamic-pituitary-adrenal axis: maternal stress activates cortisol release, which crosses the placental barrier and acts on fetal brain development through glucocorticoid receptors expressed in regions critical for sensory and emotional regulation, including the thalamus, which serves as the brain’s primary sensory relay center routing information to cortical processing areas; the hippocampus, which modulates contextual stress responses and contains particularly high glucocorticoid receptor density from midgestation onward; and the prefrontal cortex, which coordinates top-down regulation of arousal and sensory gating (Lautarescu et al., 2020; Krontira et al., 2020). A meta-analysis of population-based studies confirmed that prenatal maternal stress is significantly associated with increased risk of both autism spectrum conditions (pooled OR 1.64) and ADHD (pooled OR 1.72) in offspring (Beversdorf et al., 2019). The effects are not uniform: gestational timing, stress type, fetal sex, and genetic moderators all shape their magnitude. Family studies indicate that siblings of autistic individuals and individuals with ADHD show intermediate sensory profiles, suggesting a familial dimension to sensory processing differences that is not fully accounted for by shared genetics alone (Neufeld et al., 2021; Ghirardi et al., 2017).

The epigenetic dimension adds specificity. Oberlander et al. (2008) demonstrated that prenatal exposure to maternal depressed and anxious mood in the third trimester was associated with increased methylation of the NR3C1 glucocorticoid receptor gene in newborns, and that this methylation predicted elevated cortisol stress reactivity at 3 months of age, independently of postnatal maternal mood. This establishes a molecular pathway through which the mother’s psychological state is written into the infant’s stress regulation system before birth. Palma-Gudiel et al. (2015) confirmed the association in a meta-analysis of 977 individuals, identifying a significant correlation between prenatal stress and NR3C1 promoter methylation. The organism’s regulatory capacity is not merely shaped by postnatal experience. It is, in part, prenatally inscribed. Complementary neurobiological evidence confirms the structural consequences of this inscription: higher maternal cortisol levels in early gestation have been prospectively associated with larger amygdala volume and increased affective problems in children at age seven, independently of postnatal confounders (Buss et al., 2012). A recent perspective review further confirms that prenatal, rather than postpartum, maternal depression is the more consistent predictor of offspring neurocognitive outcomes, with effects mediated through HPA axis dysregulation and epigenetic changes in glucocorticoid receptor expression in the fetal prefrontal cortex (Severo et al., 2023).

It is important to note that maternal psychological stress constitutes one among a range of prenatal environmental factors documented to influence fetal sensory regulatory development. Maternal inflammation arising from conditions including obesity, gestational diabetes, infection, or autoimmune disease; nutritional deficiencies; obstetric complications; and exposure to environmental toxicants have all been associated with altered neurodevelopmental trajectories in offspring, including sensory processing differences (Han et al., 2021; Butler et al., 2024; Szczepara-Fabian et al., 2025). The present framework does not privilege psychological stress as a primary etiological factor; rather, it proposes that the range of conditions capable of disrupting the prenatal sensory environment may be understood, from a psychoanalytic perspective, as variations of a shared process: the transmission to the developing organism of unmetabolized excitation that the maternal system cannot adequately contain or transform. In Bionian terms, these diverse prenatal adversities may be read as beta elements operating at the physiological level, raw and unprocessed stimulation that shapes the organism’s regulatory substrate before the conditions for psychic elaboration have come into existence.

This bidirectional logic has a direct implication for prenatal intervention. If the mother’s unprocessed beta elements shape the fetal sensory substrate through the mechanisms described, then the converse also holds: to the extent that the mother develops greater capacity to metabolize and transform her own experience during pregnancy, through psychotherapeutic support, adequate relational holding, or other containing conditions, the prenatal field shared with the developing organism is correspondingly altered. The pregnant woman’s growing alpha function is, in this framework, not only her own psychic achievement but a developmental resource for the organism she carries. This reading provides a theoretical basis for prioritizing prenatal psychological support not as a secondary measure but as a primary developmental intervention.

These findings bring us to a question that empirical research cannot fully address: What psychoanalytic framework might account for the relational dimension of this prenatal inscription? We do not suggest that the fetus possesses subjectivity in the sense available to the postnatal infant. The proposal here is more circumscribed: that the prenatal period constitutes the earliest layer of what Bion (1962) termed the substrate for alpha function, understood as the capacity to process raw sensory and emotional experience into forms available for psychic organization. Before the self-other distinction exists, the organism is already being shaped by the quality of its sensory environment. That environment is not neutral: it is constituted by the mother’s physiological state: her rhythms, her cortisol levels, her movement patterns. These are conditions that act on the developing organism before the relational apparatus through which they might later be psychically processed has come into existence.

Bion’s account of the container and the contained (Bion, 1962, 1963), a concept that extends his earlier notion of the protomental matrix in which physical and mental experience remain undifferentiated (Bion, 1959; see also Ogden, 2008), describes a dyadic process in which the infant’s unprocessed beta elements (raw, unmetabolized sensory and emotional impressions) are projected into the maternal object, transformed through the mother’s reverie, and returned in a form the infant can use. The prenatal period, however, predates the conditions this model presupposes. Projection requires a self from which to project; containment requires a container experienced as other. Neither has yet differentiated. What the epigenetic and neuroendocrine literature documents, namely the direct transmission of maternal stress physiology into fetal regulatory systems, may be understood, from a psychoanalytic perspective, as a pre-objectal form of influence: the mother’s psychological state shaping the organism’s developing regulatory substrate not through relational exchange but through shared physiological conditions. This reading remains speculative in its psychoanalytic formulation; its value lies in the questions it opens rather than the mechanisms it confirms.

This pre-objectal form of influence finds clinical articulation in Debray’s (1991, 2005) work on early maternal function in psychosomatic development. Debray proposed that the mother serves as a regulatory envelope for the infant, a pare-excitation or excitation shield whose function is to filter incoming sensory and affective stimulation before it reaches the infant’s still undifferentiated psyche. When this filtering function is absent or insufficient, as in conditions of maternal depression, trauma, or psychosocial overload, the infant cannot develop adequate symbolic elaboration and instead discharges unprocessed experience through the body (Debray, 1991). This formulation extends the developmental reach of the present argument: the prenatal conditions described above, including elevated maternal cortisol and unprocessed affective material that resurfaces during pregnancy, may be understood as early determinants of the excitation-shielding capacity that the infant will depend on after birth. Marty (1958) had already proposed a biological substrate for this continuity in his account of the allergic object relation, describing an archaic fusional fixation between fetus and mother organized at a physiological level prior to any differentiated object relation, such that early immunological reactivity may reflect the relational conditions of this first undifferentiated state.

The concept of psychic transparency (*transparence psychique*), proposed by Bydlowski (2001), extends this line of thinking by attending to the maternal side of this period. Bydlowski describes a distinctive mental state of pregnancy in which the usual defenses against unconscious material are lowered, and archaic memories, preconscious fantasies, and infantile representations resurface with unusual ease. Psychic transparency has since received empirical support: Bazan et al. (2019) demonstrated a significant increase in primary process mentation in pregnant women compared to non-pregnant controls and future fathers, confirming that the pregnant woman’s psychic state is measurably, not merely metaphorically, altered. If the mother’s own unprocessed material, including material that was never adequately contained in her own early development, rises more readily to the surface during pregnancy, and if this material is expressed in the physiological conditions that constitute the fetal sensory environment, then psychic transparency may provide a psychoanalytic account of one pathway through which unmetabolized experience moves across generations (that is, affective and physiological states that were never adequately processed or transformed by the mother’s psychic apparatus are transmitted to the developing organism through the shared prenatal field, before the infant has the capacity to differentiate or elaborate them). This connection between Bydlowski’s clinical observation and the epigenetic findings of Oberlander et al. (2008) is proposed here as a conceptual bridge, not a demonstrated mechanism.

Bydlowski consistently emphasized that psychic transparency is not only a vulnerability but a developmental opportunity. When the pregnant woman has access to a sufficiently containing relational context, whether through psychoanalytic treatment, a supportive therapeutic relationship, or an adequately holding social environment, the archaic material that surfaces during pregnancy can be metabolized for the first time, interrupting rather than perpetuating the intergenerational transmission of unprocessed beta elements (Bydlowski, 2001; Bydlowski and Golse, 2001). This observation carries direct clinical implications: the prenatal period may represent a window of particular receptivity for psychotherapeutic work, precisely because the defenses that ordinarily foreclose access to early experience have temporarily receded. Intervening during pregnancy, rather than waiting for postnatal difficulties to emerge, may therefore constitute the earliest and most efficient point of entry for interrupting the developmental trajectories described in this paper.

This observation carries a further developmental implication for female offspring in particular. The oocytes that will eventually give rise to the next generation are already present in the female fetus during gestation, meaning that a pregnant woman’s prenatal environment simultaneously shapes three generations: herself as the gestating grandmother, the female fetus she carries, and the germline cells within that fetus that will become the subsequent generation’s oocytes. The epigenetic programming of these germ cells during fetal development constitutes a literal biological substrate for intergenerational transmission, one that extends the reach of the present argument beyond the dyad of mother and infant to encompass the multigenerational continuity of unprocessed experience (Bale, 2015).

The prenatal period thus emerges, from both empirical and psychoanalytic perspectives, as a formative context for the sensory regulatory substrate. Anzieu’s (1985) concept of the skin ego adds a further dimension to this picture. For Anzieu, the skin is the primary psychic envelope, the surface on which the earliest distinction between inside and outside, self and world, is inscribed through tactile experience with the caregiver. *In utero*, this relational differentiation does not yet exist. The intrauterine environment provides continuous tactile and proprioceptive stimulation, through the amniotic fluid, the uterine wall, and the fetus’s own movements, but without the intersubjective contact that would constitute a skin ego in Anzieu’s sense. The prenatal period is, in this framework, the period in which the sensory substrate on which skin ego formation will depend is itself being established, under conditions of close dependence on the mother’s physiological and psychological state.

The sensory processing differences observable across autism, ADHD, and PTSD may thus be understood, in part, as expressions of this prenatal layer, as variations in a regulatory substrate organized before birth, under conditions that shaped the organism’s subsequent capacity to filter, modulate, and integrate stimulation. The following section considers how these developmental conditions manifest in the self-soothing behaviors that clinicians across disciplines encounter in these populations.

## Self-soothing as proto-alpha: a neuropsychoanalytic reading of sensory regulation

Clinicians working with autism, ADHD, and PTSD encounter a category of behavior that resists easy classification. The autistic individual who rocks rhythmically in moments of overwhelm; the adult with ADHD who taps, fidgets, or seeks high-intensity sensory input to sustain concentration; the trauma survivor who dissociates, freezes, or compulsively repeats physical routines when triggered: these behaviors are phenomenologically diverse, yet they share a common structure. They are repetitive, non-symbolic, and appear to serve a regulatory function that operates below the threshold of intentional self-reflection. Clinical practice has tended to frame them as symptoms to be reduced or eliminated. This analysis suggests that such framing may obscure their functional significance, and that a psychoanalytic reading of their developmental origins offers a more generative perspective.

The French psychosomatic tradition, and Claude Smadja’s (1993) conceptualization of *procédés auto-calmants* in particular, provides theoretical vocabulary for this reading. The concept of *procédés auto-calmants* was first introduced by Fain (1971) within the Paris Psychosomatic School, through clinical observations of infants whose mothers employed repetitive rocking as a soothing strategy in the absence of genuine relational containment. Smadja (1993) subsequently elaborated the concept into a formal clinical framework. Smadja defines auto-soothing procedures as motor or perceptual activities that the ego deploys to counter-invest a traumatic reality threatening its integrity, activities aimed not at satisfaction but at the restoration of calm. He distinguishes these procedures sharply from symptoms in the psychoanalytic sense: a symptom arises from conflict and produces a compromise formation carrying unconscious meaning; a self-soothing procedure, by contrast, operates in the register of discharge rather than symbolization, seeking to evacuate excitation that cannot be mentally processed. It produces not satisfaction but a temporary reduction in arousal, a return to quiet rather than a movement toward desire (Smadja, 1993; İkiz, 2005).

This pattern has particular developmental relevance. As İkiz (2005) demonstrates in the context of mother-infant relations, when the maternal object fails to provide adequate soothing containment, the infant learns to regulate excitation through motor activity rather than psychic elaboration. A pattern once established in this way tends to persist into adulthood as an automatic regulatory strategy deployed in the absence of symbolic elaboration. This observation connects the clinical phenomenology of self-soothing behaviors directly to the developmental and relational conditions described in the preceding section, where prenatal stress and insufficient early containment are proposed as factors shaping the organism’s regulatory substrate from the earliest stages of development.

This distinction maps onto Bion’s (1962) framework with considerable precision. Where mental elaboration is possible, beta elements (raw, unmetabolized sensory and affective impressions) are transformed through alpha function into alpha elements available for dreaming, thinking, and symbolic representation. Where alpha function is unavailable or insufficient, beta elements cannot be processed: they accumulate, press for evacuation, and find their discharge through action rather than thought. Self-soothing behaviors, read in this light, are not failures of self-regulation but evidence of the organism’s attempt to manage beta element pressure (the build-up of unprocessed sensory and affective excitation that, in the absence of symbolic elaboration, presses for discharge through action rather than thought) through the only channels available: motor activity, rhythmic stimulation, proprioceptive input. They are, in Bion’s terms, the work of a psyche that cannot yet dream.

Tustin’s (1980, 1984, 1986) clinical work offers a complementary perspective, particularly relevant to the tactile and proprioceptive dimensions of these behaviors. Tustin distinguished between autistic objects (hard external objects used to generate sensations of bodily boundedness and strength) and autistic shapes: soft, endogenously produced sensations arising from the body’s own surfaces, movements, and substances, serving as what she described as “a self-induced warm bath that is always on tap” (Tustin, 1986, p. 128). Although Tustin developed these concepts in the context of childhood autism, she explicitly extended their applicability to neurotic and adult presentations (Tustin, 1986), and subsequent clinical literature has documented their relevance across presentations in which early uncontained sensory experience has been silently encapsulated. The stimming behaviors of autistic adults, the fidgeting and movement-seeking of adults with ADHD, and the somatic rituals of trauma survivors share structural features with Tustin’s autistic shapes: they are sensation-producing, self-generated, repetitive, and oriented not toward an object but toward the restoration of a felt bodily boundary.

Anzieu’s (1985) concept of the skin ego deepens this reading, drawn upon here not as an etiological account of neurodevelopmental atypicality but as a theoretical framework for understanding the developmental history of bodily self-organization. For Anzieu, the skin ego encompasses multiple psychic functions: it serves as a containing envelope holding psychic contents together, as a screen filtering external stimulation, as a surface of communication between inner and outer worlds, and as a boundary establishing the distinction between self and not-self. These functions are not reducible to tactile contact alone but emerge from the full sensory matrix of early caregiving experience, including vocal holding, thermal regulation, and rhythmic attunement. For Anzieu, the skin functions not only as the physical boundary of the body but as the primary psychic envelope, the surface on which the earliest distinction between inside and outside, self and world, is established through the cumulative sensory experience of early caregiving. When this envelope fails to consolidate as a result of insufficient, unpredictable, or overwhelmingly aversive early tactile experience, the individual may spend a lifetime seeking external substitutes for the containing function the skin ego did not adequately develop. Deep pressure, weighted blankets, the rhythmic friction of stimming, the proprioceptive input of vigorous exercise: from an Anzieuian perspective, these are attempts to construct through repeated sensory experience what was not sufficiently established through early relational contact. When read alongside the argument developed in the preceding section, these behaviors may be understood more precisely as compensatory mechanisms: lifelong attempts to establish, through repetitive bodily activity, the sensory boundary and regulatory capacity that the skin ego did not adequately develop, itself a consequence of a prenatal sensory substrate that was insufficiently prepared by the conditions of early gestation.

Drawing on Smadja’s auto-soothing procedures, Bion’s beta element processing, and Tustin’s autistic shapes and objects, we propose the term proto-alpha mechanisms to describe this functional category: bodily procedures that perform, in the somatic register, an approximation of the psychic work that alpha function performs in the mental register. Proto-alpha mechanisms do not symbolize; they regulate. They do not represent; they contain. In populations whose capacity for mental elaboration of sensory experience has been compromised, whether through neurodevelopmental organization, traumatic inscription, or prenatal substrate, these mechanisms may constitute the organism’s most available means of maintaining psychic coherence.

The term proto-alpha is chosen deliberately. In Bion’s account, alpha function is a relational achievement: it presupposes a containing object, a dyadic space in which unprocessed experience can be metabolized and returned in a form the psyche can use. Proto-alpha mechanisms, by contrast, operate without this relational structure, either because it has not yet come into existence, as in the prenatal period, or because it was never adequately established, leaving the organism to manage beta element pressure through the body alone. They are not failures of alpha function but its precursors and somatic substitutes: the organism’s attempt to perform in the bodily register what alpha function performs in the relational and mental register. This distinction has a direct clinical implication: treating proto-alpha mechanisms as symptoms to be eliminated, without first establishing the relational conditions under which genuine alpha function can develop, is likely to produce symptomatic substitution rather than genuine regulatory growth.

If self-soothing behaviors across autism, ADHD, and PTSD share this functional structure, then the surface diversity of their forms reflects different developmental pathways leading to a common regulatory response, rather than different underlying processes. The autistic individual’s rocking, the ADHD adult’s sensory seeking, and the trauma survivor’s somatic rituals are, at this level of analysis, variations of a single response: the organism attempting to process through its body what it cannot process through its mind.

## Discussion

This analysis has approached three diagnostically distinct conditions through a shared developmental lens, proposing that sensory processing differences constitute a transdiagnostic dimension linking autism, ADHD, and PTSD at the level of developmental organization. The prenatal period, we have suggested, is not merely a biological antecedent to this dimension but its earliest relational context: the intrauterine environment in which the sensory systems that will later show characteristic atypicalities first become functional, and in which the mother’s psychological state, mediated through HPA axis activation and glucocorticoid receptor epigenetics, constitutes the organism’s first sensory environment (Lautarescu et al., 2020; Oberlander et al., 2008). The psychoanalytic frameworks drawn upon, namely Bion’s (1962) theory of alpha function and containment, Smadja’s (1993) conceptualization of auto-soothing procedures, Tustin’s (1980, 1984, 1986) work on autistic objects and shapes, and Anzieu’s (1985) skin ego, offer a language for what the empirical findings point toward but cannot fully articulate: the relational texture of early sensory experience, and the functional meaning of the regulatory behaviors that persist when that experience has been insufficient.

It is important to clarify a distinction that bears directly on the clinical implications proposed here. Neurodiversity, understood as the neurological organization that gives rise to the sensory processing differences described in this paper, is not proposed as a target for therapeutic alteration. Contemporary neurodiversity-affirming frameworks explicitly caution against the goal of normalizing or eliminating the neurodivergent neurobiological substrate, arguing instead for support, environmental adaptation, and identity-affirming approaches (Chapman and Botha, 2022; Pantazakos, 2023). The framework offered here aligns with this position: no claim is made that psychoanalytic or any other form of therapy has the capacity to modify the underlying neurological organization. What is proposed is more circumscribed: that the secondary adaptive difficulties arising from neurodivergent sensory organization, including the reliance on proto-alpha mechanisms as primary regulatory strategies, may be addressed through interventions that offer the relational containment in which symbolic elaboration becomes gradually possible. Therapy, in this reading, does not change the neurodiversity substrate; it may create the conditions in which the organism can make fuller use of the regulatory capacity it already possesses.

The clinical implications of this framework differ depending on the practitioner’s discipline, though a common reorientation underlies them. For the clinician working psychoanalytically or psychodynamically, the framework invites specific caution in how self-soothing behaviors are encountered in the clinical setting. Rather than interpreting them prematurely as defenses, resistances, or enactments (readings that presuppose a symbolic register that may not yet be available), the clinician may first consider them as proto-alpha mechanisms: the patient’s most available means of maintaining psychic coherence. Tustin (1986) observed that the analyst’s premature interpretation of autistic maneuvers often disrupts the fragile regulatory function they serve. The same caution extends further: before asking what a self-soothing behavior means, it is worth asking what it does, and whether the therapeutic relationship yet offers sufficient containment to make its abandonment safe.

A parallel reorientation applies to clinicians working somatically, behaviorally, or through occupational therapy. Sensory integration interventions targeting processing differences without attending to their developmental and relational origins may achieve symptomatic improvement while leaving the underlying organizational deficit unaddressed. Conversely, sensory-based interventions offered within a sufficiently containing relational frame may operate through mechanisms more profound than the purely neurological. The therapeutic relationship is itself a sensory environment: its rhythms, its predictability, its attunement to the patient’s arousal states constitute a form of containment that Bion (1962), Anzieu (1985), and the French psychosomatic school would recognize as the relational precondition for the development of alpha function.

For the psychiatrist or general practitioner, the framework suggests the value of including a developmental sensory history in clinical evaluation. Before a diagnosis is assigned and pharmacological intervention initiated, the question of how this individual has experienced and regulated sensory stimulation across the lifespan, and what can be inferred about the conditions of their earliest sensory organization, may constitute clinically relevant information. Consider the adult presenting with attentional dysregulation, emotional lability, and sensory overreactivity who receives an ADHD diagnosis and begins stimulant medication. If the underlying organization reflects a neurodevelopmental sensory processing difference, stimulant medication may produce meaningful improvement. If the same clinical picture reflects a traumatic organization of the nervous system, stimulant medication may intensify rather than reduce the dysregulation (Wendt et al., 2023). The phenotype is identical; the etiology differs; and the etiology, not the phenotype, may better guide intervention.

Beyond the diagnostic evaluation, the framework proposed here invites a broader reconceptualization of the clinical anamnesis: systematically including the prenatal relational and psychological history as a routine dimension of assessment may open interpretive possibilities that categorical diagnosis forecloses. Information about the mother’s psychological state during pregnancy, the availability of relational support, and the quality of the early holding environment constitutes clinically relevant context for understanding not only how a condition manifests but how it came to be organized in this particular person.

Across these disciplinary distinctions, a common clinical orientation emerges: attending to the prenatal relational matrix as a constitutive dimension of sensory regulatory development opens a different kind of clinical listening, one that hears behind the symptom or the diagnostic category a prenatal developmental history that preceded language, relationality, and the self-other distinction itself.

This conceptual analysis also opens several empirically testable directions. If prenatal maternal stress constitutes a vulnerability factor for sensory processing differences, operating through the mechanisms documented by Lautarescu et al. (2020), Oberlander et al. (2008), and Palma-Gudiel et al. (2015), then individuals with autism, ADHD, and PTSD might be expected to show elevated rates of prenatal stress exposure relative to controls, with this association partially mediated by neonatal sensory reactivity measures. If sibling similarity in sensory profiles reflects shared prenatal conditions rather than solely shared genetics, as familial studies suggest (Neufeld et al., 2021; Ghirardi et al., 2017), then sensory processing similarity in siblings may be more strongly predicted by measures of maternal psychological organization during pregnancy than by genetic relatedness alone. If the self-soothing behaviors observed in these populations constitute proto-alpha mechanisms serving equivalent regulatory functions, their reduction through therapeutic intervention might be expected to occur alongside, rather than instead of, the development of symbolic and relational elaboration capacity.

The sensory dysregulation documented across these three populations may also be understood within the Free Energy Principle framework (Friston, 2010), which conceptualizes perception as active inference and frames sensory dysregulation as a failure of precision-weighted prediction error minimization. From this perspective, the organism does not passively receive sensory input but continuously generates predictions about incoming stimulation, updating these predictions in response to error signals. In autism, ADHD, and PTSD, this prediction-updating system operates with atypical precision weighting, producing the characteristic patterns of hyper- and hyposensitivity documented in the empirical literature. The present framework addresses a question that computational accounts leave open: Why is the prediction system calibrated in this way? We propose that the prenatal conditions described here, including elevated maternal cortisol, epigenetic programming of stress reactivity, and the psychic climate of pregnancy as conceptualized by Bydlowski (2001), constitute early determinants of the organism’s baseline prediction error threshold, shaping the system before postnatal experience begins. The psychoanalytic framework proposed here is therefore not an alternative to computational models such as the Free Energy Principle but an account of the relational and developmental substrate in which the prediction system is first organized.

The prenatal hypothesis developed here holds that the mother’s psychological state during pregnancy shapes the fetal sensory regulatory substrate through documented neuroendocrine and epigenetic pathways, and that psychoanalytic concepts may offer complementary accounts of the relational dimension of this process. It is offered as a conceptual framework for further investigation, not as a confirmed mechanism. Its psychoanalytic elaborations remain speculative where empirical support is absent, and are presented as such.

What this analysis hopes to demonstrate is that the meeting point between contemporary neuroscience and psychoanalytic theory is not merely intellectually productive – it is clinically necessary. A clinician who understands sensory dysregulation only through the lens of neurobiology may miss the developmental history that shaped it. A clinician who understands it only through the lens of psychoanalytic object relations may miss the biological substrate on which that history was inscribed. The framework examined here is an attempt to hold both – not by reducing one to the other, but by reading each in the light of what the other makes visible.

The child who rocked to sleep alone in a corner, the adult who cannot tolerate an open-plan office, the trauma survivor whose body still scans for threat in a room that is safe: these are not three different problems requiring three different solutions. They are three expressions of an organism whose earliest sensory world did not provide what was needed for the development of a self-regulating, containing, symbolizing psyche. Understanding that is the beginning of helping them.
